# Trends in Spontaneous Cerebrospinal Fluid Leak Repairs in the United States, 2009–2018

**DOI:** 10.1097/ONO.0000000000000021

**Published:** 2022-10-27

**Authors:** Douglas J. Totten, Elizabeth Schueth, Mohamad Z. Saltagi, Cyrus Rabbani, Alyssa Hartsell Harris, Dani Tressman, Samuel F. Hohmann, Rick F. Nelson

**Affiliations:** 1 Department of Otolaryngology—Head and Neck Surgery, Indiana University, School of Medicine, Indianapolis, IN; 2 Indiana University School of Medicine, Indianapolis, IN; 3 Vizient Inc., Chicago, IL; 4 Department of Health Systems Management, Rush University, Chicago, IL; 5 Department of Neurosurgery, Indiana University, School of Medicine, Indianapolis, IN.

**Keywords:** Cerebrospinal fluid, Craniotomy, CSF leak, Spontaneous

## Abstract

**Background:**

Rates of spontaneous cerebrospinal fluid leak (sCSF) repairs have increased in recent decades in line with increases in obesity rates.

**Objectives:**

To determine if the national rate of sCSF leak has continued to rise in recent years and to identify associated risk factors utilizing a comprehensive national database comprising most academic medical centers.

**Methods:**

A retrospective review from 2009 to 2018 was performed using the Vizient Clinical Database (CDB) of 105 leading academic medical centers in the United States. Patients who underwent CSF leak repair in the CDB database using *ICD-9* and *ICD-10* diagnostic and procedure codes. Patients with epidural hematomas over the same time frame were used as a control. National rates of craniotomy for sCSF leak repair each quarter were assessed and sCSF leak patient characteristics (age, gender, obesity, hypertension, diabetes) were calculated.

**Results:**

The rate of craniotomy for all sCSF leak repairs increased by 10.2% annually from 2009 to 2015 (*P* < 0.0001). There was no statistically significant change in the rate of epidural hematomas over the same period. The rate of lateral sCSF leak repair increased on average by 10.4% annually from 2009 (218 cases/year) to 2018 (457 cases/year) (*P* < 0.0001). A statistically significant increase was observed across all regions of the United States (*P* ≤ 0.005). sCSF leak patients had an average (standard deviation) age of 55.0 (13.2) years and 67.2% were female. Obesity was the only demographic factors that increased significantly over time. Likely due to comorbid factors, Black patients comprise a disproportionately large percentage of lateral sCSF leak repair patients.

**Conclusions:**

The rate of craniotomy for spontaneous CSF leaks continues to rise by approximately 10% annually.

Cerebral spinal fluid (CSF) leaks typically occur secondary to trauma, surgery, tumors, infection, or a congenital abnormality (1,2). Spontaneous cerebrospinal fluid (sCSF) leaks occur in the absence of any of these known etiologies (2). By definition, sCSF leaks occur secondary to dural defects and defects in the skull base over the middle ear, mastoid, and/or pneumatized sinuses anteriorly (3). Due to intracranial communication, patients with sCSF leaks are at increased risk of intracranial infection and about 19% develop meningitis (4). Spontaneous CSF leaks do not resolve spontaneously and typically require surgical repair of the skull base and dura.

Spontaneous CSF leaks are highly associated with obesity, as approximately 70%–80% of sCSF patients are obese (5–11). The prevalence of obesity in the United States has nearly tripled since 1975 (12–14). Currently, it is estimated 39% of adults in the United States are obese with some observed racial predilections (15–17). The development of a spontaneous CSF leak likely takes years, from the development of obesity and comorbid conditions to skull thinning and eventual CSF leakage into pneumatized sinuses (7). Prior research has shown that national sCSF leak repair rates have doubled from 2002 to 2012, have increased with increasing obesity rates, and are most common in regions with the higher obesity rates (7).

This study aimed to determine if both total and lateral sCSF leak repair rates have continued to rise nationwide from 2009 to 2018. We also aimed to determine if there are racial differences in lateral sCSF leak repair incidence and if rates of obesity, hypertension, or other comorbidities are significantly contributing to rising rates of lateral sCSF leaks. To control for possible confounding factors, such as increased hospital volume, the rate of epidural hematomas over time was also examined, as the rate of this diagnosis was not expected to have significantly changed over the examined time frame.

## METHODS

### Data Source

Vizient is a member-owned corporation that includes 97% of academic medical centers (AMCs) as well as other medical institutions. The Clinical Data Base and Resource Manager (CDB/RM) are comprised of clinical, financial, and administrative data reported from each institution for purposes of research, comparison, and quality improvement. The Vizient CDB/RM consists of leading AMCs, complex teaching, and community hospitals across the United States. A total of 160 medical centers fully reported data for all quarters included in this study with 105 medical centers reporting at least 1 sCSF leak repair.

This resource was searched using *International Statistical Classification of Diseases and Related Health Problem*s (*ICD-9* and *ICD-10*) diagnosis and procedure codes. This retrospective review of the Vizient (CDB/RM) used de-identified data on all patients who underwent a craniotomy for sCSF leak repairs between October 2009 and September 2018. Importantly, diagnosis and procedure coding transitioned from *ICD-9* to *ICD-10* in October 2015, which resulted in a significant expansion of potential *ICD-10* codes. Attempts were made to crosswalk both diagnostic and procedure codes from ICD-9 to *ICD-10* for both sCSF leaks and epidural hematomas. CSF rhinorrhea (*ICD-9*: 349.81), CSF otorrhea (*ICD-9*: 388.61), and encephalocele (*ICD-9*: 7420) were converted to CSF Leak (*ICD-10*: G96.0), encephalocele of other site (*ICD-10*: Q01.8), encephalocele, unspecified (*ICD-10*: Q01.9). A 33% drop in the total number of cases was observed when transitioning to ICD-10 coding after Q3 of 2015. When the CSF rhinorrhea (*ICD-9*: 349.81) code was removed from 2009 to 2015, this drop in cases was not observed suggesting that *ICD-10* codes for repair of CSF rhinorrhea were not cross walked from *ICD-9.* Multiple attempts were made to resolve this issue without success. Thus, we analyzed both total sCSF leaks and lateral sCSF leaks using *ICD-9* diagnostic and procedure codes from 2009 to Q3 2015. After the conversion to *ICD-10*, we limited our analysis to just lateral sCSF leaks. Institutional review board and patient informed consent was not required as all patient data in the Vizient CDB/RM is de-identified.

Patients were identified using *ICD-9* diagnosis codes of 388.61 (cerebrospinal fluid otorrhea) and 7420 (encephalocele)—and, for all sCSF leaks, 349.81 (cerebrospinal fluid rhinorrhea)—and *ICD-9* procedure codes of 021 (repair of cerebral meninges), 0211 (simple suture of dura mater of brain), and 0212 (other repair of cerebral meninges). After conversion to *ICD-10* in October 2015, patients were identified with the following primary *ICD-10* diagnostic codes: G96.0 (cerebrospinal fluid leak), Q01.8 (encephalocele of other sites), and Q01.9 (encephalocele, unspecified) along with primary *ICD-10* procedure codes. To ensure we captured all relevant repair procedure codes, we searched for all *ICD-10* procedures codes linked to the G96.0, Q01.8, and Q01.9 diagnostic codes in 2017. The following *ICD-10* repair procedure codes were the top 25 codes and were chosen in our search for sCSF leaks using the above primary diagnostic code and these primary procedure codes: 00Q10ZZ (repair cerebral meninges, open), 00Q13ZZ (repair cerebral meninges, percutaneous), 00Q14ZZ (repair cerebral meninges, percutaneous endoscopic), 00Q20ZZ (repair dura matter, open), 00Q23ZZ (repair dura matter, percutaneous), 00Q24ZZ (repair dura matter, percutaneous endoscopic), 00U107Z (supplement cerebral meninges with autologous, open), 00U10JZ (supplement cerebral meninges with synthetic, open), 00U10KZ (supplement cerebral meninges with nonautologous, open), 00U207Z (supplement dura mater with autologous, open), 00U20JZ (supplement dura mater with synthetic, open), 00U20KZ (supplement dura mater with nonautologous, open), 00U237Z (supplement dura mater with autologous, percutaneous), 00U24JZ (supplement dura mater with synthetic, endoscopic), 00U24KZ (supplement dura mater, nonautologous, percutaneous endoscopic). To obtain spontaneous CSF leaks patients, we also excluded patients with secondary procedure code (*ICD-9*, *ICD-10*): excision of acoustic neuroma (0401, 00BN0ZZ), excision of pituitary gland, transsphenoidal (0762, 0GT04ZZ), and codes related to skull or skull base fractures (eg, 80100, S0210XA). Patients under 18 years of age were excluded. Demographic and co-morbid data for each patient were collected, including sex, age, race, obesity, hypertension, and diabetes. Body mass index (BMI) data were not available. Region of reporting medical center was also recorded.

Diagnosis of epidural hematomas was used as a control variable across a similar time frame (2009 Q4 through 2015 Q3). Patients were identified using *ICD-9* diagnosis code 8524 (hematoma, extradural or epidural). Attempts were made to crosswalk *ICD-9* data with *ICD-10* data using *ICD-10* diagnosis codes of S064 (epidural hemorrhage) and I621 (nontraumatic extradural hemorrhage). However, due to substantial reporting inconsistencies across this transition, the control cohort was limited to *ICD-9* reports. Additionally, due to difficulties with this crosswalk and with coordinating diagnosis codes with procedure codes for craniotomy, analysis was limited to the diagnosis of epidural hematoma.

### Statistical Analysis

Vizient data were collected with the help of A.H., S.H., and D.Tr. Descriptive and analytic statistics were calculated using Excel and SAS software (SAS Institute, Cary, NC). The change in population variables by advancing quarter, including average age, rates of obesity, hypertension, and diabetes, sex percentages, and racial percentages, were also assessed using single-predictor linear regression. Correlation between population variables and sCSF leak rates were assessed using Pearson Correlation. Multivariable linear regression model was used to assess change in quarterly sCSF leak repairs and possible predictor variables with significant changes over this time period or possible correlation with sCSF leak rates to assess impact of predictors on rate of repairs. Regional change in sCSF leaks over time was also assessed using single-predictor linear regression for each of the 4 national regions (Northeast, Midwest, South, West) based on the 2010 US Census regional distribution map. Single-predictor linear regression was used to assess change in the rate of epidural hematomas at reporting hospitals from 2009 Q4 to 2015 Q3. Pearson Correlation was used to assess correlation between epidural hematoma and sCSF leak rates from 2009 Q4 to 2015 Q3.

## RESULTS

A total of 4743 patients underwent craniotomy (including both lateral and anterior skull base repair) for sCSF leak from 2009 to 2015. Mean age of all patients undergoing craniotomy for sCSF leak was 53.9 (standard deviation [SD]: 13.5). Consistent with previous reports, females comprised the majority of patients (3270 [68.9%]) (7). Hypertension was diagnosed in 2617 (55.2%) of patients, obesity in 1430 (30.1%), diabetes in 1139 (24.0%), and obstructive sleep apnea (OSA) in 893 (18.8%). A total of 3155 (66.5%) were White compared to 995 (20.9%) Black patients and 47 (1.0%) Asian patients while 264 (5.6%) of patients identified as having Hispanic ethnicity. Race was recorded as other or was unknown in 546 (11.5%) of patients. The greatest number of cases were observed in the South (1937 [40.8%]) followed by the Midwest (1354 [28.5%]), the Northeast (826 [17.4%]), and the West (607 [12.8%]).

**FIG. 1.**
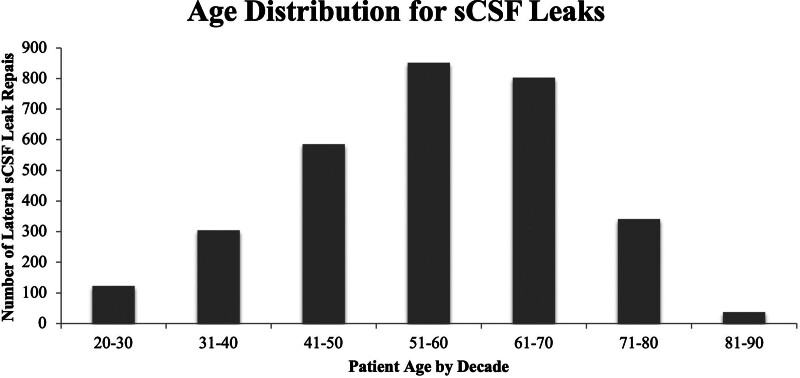
Distribution of age of patients undergoing lateral sCSF leak repairs by decade. sCSF indicates spontaneous cerebrospinal fluid.

Primary analysis was performed on the cohort of patients undergoing lateral sCSF leak repair, as this cohort spanned the greatest number of years. A total of 3061 patients underwent craniotomy for lateral sCSF leak from 2009 to 2018. Mean age in this cohort was 55.0 (SD: 13.2) years (Fig. 1). Lateral sCSF leaks were also more common in females (2057 [67.2%]). A diagnosis of hypertension was recorded in 1785 (58.3%) of lateral sCSF leak patients while obesity diagnosis was recorded in 981 patients (32.0%), diabetes in 805 (26.2%), and OSA in 651 (21.3%) (Table 1). Females comprised 67.2% of the patient cohort. Racially, 69.0% sCSF leak patients were White, 20.0% were Black, and 0.8% were Asian, while race was recorded as “other” or not recorded for 10.1% of patients and 4.5% of patients identified as having Hispanic ethnicity. Black patients had higher rates of obesity (41.3%), hypertension (73.7%), diabetes (31.7%), and OSA (24.6%) than White or Asian patents (Table 2). Regionally, the number of reported sCSF leaks across this time frame was highest in the South (1229, or 40.2% of total reported sCSF leaks) followed by the Midwest (885, 28.9%), the Northeast (585, 19.1%), and the West (362, 11.8%). All tables reflect results from the lateral sCSF leak repair cohort.

**TABLE 1.** Patient demographics for lateral sCSF leak and epidural hematoma cohorts

	Lateral sCSF leak (n = 3061)	Epidural hematoma (n = 2022)
Mean age, y (SD)	55.0 (13.2)	41.5 (28.0)
Gender, n (%)		
Male	1004 (32.3)	1335 (66.0)
Female	2057 (67.2)	687 (34.0)
Comorbidities, n (%)		
HTN	1785 (58.3)	638 (31.5)
Obesity	981 (32.0)	38 (1.9)
Diabetes	805 (26.2)	215 (10.6)
Race, n (%)		
White	2113 (69.0)	1342 (66.3)
Black	613 (20.0)	206 (10.2)
Asian	25 (0.8)	70 (3.5)
Other/unspecified	310 (10.1)	405 (20.0)

n = number of patients.

sCSF indicates spontaneous cerebrospinal fluid; SD, standard deviation; HTN, hypertension.

**TABLE 2.** Lateral sCSF leak reported patient health characteristics by race

	Hypertension n (%)	Obesity n (%)	Diabetes n (%)	OSA n (%)
All patients (n = 3061)	1785 (58.3)	981 (32.1)	805 (26.3)	651 (21.3)
White (n = 2113)	1171 (55.4)	657 (31.1)	521 (24.7)	460 (21.8)
Black (n = 613)	452 (73.7)	253 (41.3)	194 (31.7)	151 (24.6)
Asian (n = 25)	9 (36.0)	3 (12.0)	6 (24.0)	3 (12.0)
Other/unknown (n = 335)	162 (48.4)	71 (21.2)	90 (26.9)	40 (11.9)

sCSF indicates spontaneous cerebrospinal fluid.

A total of 2023 patients were diagnosed with an epidural hematoma from 2009 Q4 to 2015 Q3 from 121 medical centers with at least 1 reported epidural hematoma. The mean (SD) age in the epidural hematoma cohort was 41.5 (28) years while 34.0% of epidural hematoma patients were female. White patients comprised 66.3% of this cohort while Black and Asian patients made up 10.2% and 3.5% of the total, respectively. A total of 30 (1.5%) of patients had a diagnosis of OSA, 38 (1.9%) had a diagnosis of obesity, 637 (3.1%) had a diagnosis of hypertension, and 215 (10.6%) had a diagnosis of diabetes mellitus (Table 1). The percentage of White patients in the epidural hematoma cohort (66.3%) was similar to that in the sCSF leak cohort (69.0%). Black patients comprised 20.0% of the sCSF leak repair cohort and 10.2% of the epidural hematoma cohort.

**FIG. 2.**
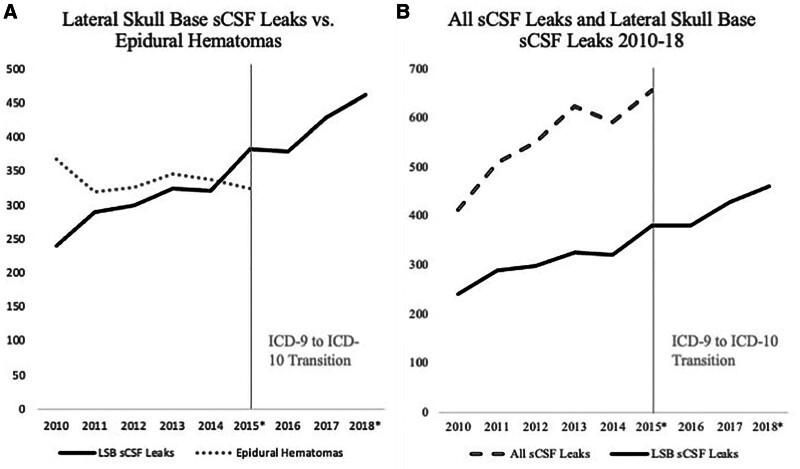
A, Lateral skull base sCSF leak repairs from 2010 to 2018 compared with the rate of epidural hematomas from 2010 to 2015. B, Lateral skull base sCSF leak repairs (2010–2018) vs all sCSF repairs (2010–2015). *In cases where there was no data for the fourth quarter of a year, the number of cases reported in the preceding year was utilized to establish an estimate of total annual cases. The year 2009 was excluded from this graph as data were only available for one quarter of this year. sCSF indicates spontaneous cerebrospinal fluid.

The rate of all sCSF leaks was assessed from 2009 Q4 to 2015 Q3. The rate of all sCSF leak repairs increased at an average rate of 10.2% annually with an overall increase of 59.7% averaged over 12 months (Fig. 2A, B; *P* < 0.0001). The rate of epidural hematomas over the same time frame decreased by 11.2% averaged over 12 months, although this change was not significant (Fig. 2A; *P* = 0.6307). There was no significant association between rate of all sCSF leak repairs and rate of epidural hematomas (*P* = 0.8246).

Lateral sCSF leak cohort was subsequently examined over 9 years by removing of patients with the diagnosis of CSF rhinorrhea. A significant increase of 10.4% per year was observed in this cohort from 2009 Q4 to 2018 Q3, with an overall increase of 110% averaged over 12 months (Fig. 2A, B; *P* < 0.0001). There was no significant association between change in lateral sCSF leaks and epidural hematomas (*P* = 0.6307). Single predictor linear regression was used to assess changes in population variables and possible predictors in the sCSF cohort from 2009 Q4 to 2018 Q3 (Table 3). Obesity rate alone experienced a significant change over this time period (β = 0.003, *P* = 0.013). Pearson correlation was also used to identify possible relationships between demographic variables and latral sCSF leak repair rates (Table 3). Obesity (*r* = 0.579, *P* < 0.001) was strongly correlated with lateral sCSF leak repair rates while Black race (*r* = 0.335, *P* = 0.046) had a small-to-moderate correlation. Multivariable linear regression was subsequently performed using these variables as possible predictors for lateral sCSF leaks in addition to time. Only time (as advancing quarters) was significantly associated with increased lateral sCSF leak rates (*P* < 0.0001) (Table 4). This increase in lateral sCSF leaks was observed in each region (Table 5).

**TABLE 3.** *Patient characteristics: correlation with lateral sCSF leaks and change over time (in advancing quarters*)

	Correlation with lateral sCSF leaks		Change over time
Variable	Correlation	*P* value	*P* value
Age	0.235	0.169	0.075
Black	0.335	0.046	0.130
White	−0.204	0.233	0.145
Asian	−0.227	0.182	0.410
Male	−0.145	0.401	0.416
OSA	0.285	0.092	0.441
Obesity	0.579	<0.001	0.001
HTN	0.153	0.372	0.882
DM	0.117	0.491	0.702

DM indicates diabetes mellitus, HTN, hypertension; OSA, obstructive sleep apnea; sCSF, spontaneous cerebrospinal fluid.

**TABLE 4.** Multivariable linear regression with possible predictors for increased lateral sCSF leaks

	Estimate	Std. Error	*P* value
Time (advancing quarter)	1.4224	0.15203	<0.0001
Obesity	42.658	23.54923	0.0795
Black race	52.166	36.75731	0.1655

sCSF indicates spontaneous cerebrospinal fluid.

**TABLE 5.** Increase in lateral sCSF leaks by region each quarter from October 2009 to September 2018

Region	Estimate	Std error	*P* value
Northeast	0.2331	0.0778	0.0051
South	0.5348	0.1100	<0.0001
Midwest	0.5999	0.0930	<0.0001
West	0.2425	0.0627	0.0005

sCSF indicates spontaneous cerebrospinal fluid.

## DISCUSSION

Between October 2009 and September 2018, the number of lateral sCSF leak repairs increased by 110% in the medical centers fully reporting to Vizient over this decade. This follows a previous study showing that the rate of sCSF leak repairs had doubled in the United States from 2002 to 2012 (7). Our findings confirm that the rate of lateral sCSF leak repairs in the Unites States continues to rise rapidly. As demonstrated by Figure 2B, this trend is also true of all sCSF leak repairs as well for the data available (October 2009–September 2015). This is likely due, in part, to the persistent obesity epidemic and associated comorbid conditions (eg, OSA) linked to the development of sCSF leaks (5,18–23). It is notable, however, that time (in consecutive quarters) appears more predictive of increased lateral sCSF leak rates than changing obesity rates. It is likely that sCSF leaks take years to occur after the development of obesity and comorbid conditions, suggesting the number of sCSF leak repairs—both laterally and comprehensively—is likely to continue to increase (7). Furthermore, it is possible that not all patients fitting obese criteria (BMI ≥30.0) were captured in this study, as the obesity rate in this cohort of sCSF leak patients (32.0%) is below the national obesity rate (35.7% in 2010), contrary to what would be expected given the association between sCSF leaks and obesity (24). Additionally, improvements and possible expansion in imaging may account for a higher rate of diagnosis and, consequently, repairs. While the *ICD-10* transition makes it difficult to compare all sCSF leak repairs (including anterior skull base repairs) from 2009 to 2018, the marked increase in sCSF leak repairs were increasing rapidly from 2009 to 2015 and persistent—and consistent—increase in lateral sCSF leak repairs from 2015 to 2018 would suggest that the total rate of sCSF leak repairs continues to escalate.

The gender and age distributions of this study are similar to prior studies with 67.2% of lateral sCSF patients being female with an average age of 55.0 (13.2) years, with similar gender and age distributions among all sCSF leak patients (53.9 [13.5]) (5,11,20,25–27). While this study only captures reported data from participating institutions, the Vizient CDB/RM encompasses the vast majority of AMCs and many other medical institutions and thus likely captures majority of sCSF leak and epidural hematoma encounters. Regardless, this study demonstrates that, among the medical centers fully reporting over this time period, a significant and drastic increase in sCSF leaks has persisted well beyond what would be expected from increased patient volumes alone, as shown by the stable number of epidural hematomas over a similar time period. This increase is observed in medical centers in all four national regions while demographic variables, including race, age, and gender, do not appreciably change in this cohort over time. Consequently, the increasing number of sCSF leaks appears to be a national phenomenon that is not isolated to particular areas, populations, or people groups, even as specific population characteristics were observed.

While the majority of patients who had lateral sCSF leak repairs were White (69.0%), Black patients appear to have a higher incidence of sCSF leaks (20.0%) than would be expected based on the Black population of the United States (12.6% based on 2010 US Census data). This is likely due to high rates of underlying health condition, such as obesity, hypertension, and diabetes, among Black patients (28,29). This is further reflected in the rate of comorbid diagnoses across race in this cohort (Table 2). Obesity-associated co-morbid conditions (OSA and idiopathic intracranial hypertension [IIH]) are known to increase intracranial pressure both transiently or chronically and disproportionately affect the Black population (5,18–21,28–34). Furthermore, both OSA and IIH are independently associated with global calvarial and skull base thinning over time and have been shown to be more common in sCSF leak patients than the general population (5,18–20,22,34–36). Thus, while Black patients comprise a disproportionately high percentage of lateral sCSF leak patients in this cohort, this finding is likely explained, in whole or in part, by underlying health factors. Furthermore, prior research has found that regions with higher obesity rates had twice the rate of sCSF leak repairs as regions with lower obesity rates (7). While not necessarily comprehensive, our data suggest this finding continues to be accurate, with the number of sCSF leaks is continuing to rise in all 4 national regions.

### Limitations

This study reflects the data reported by 105 medical centers which may not be entirely representative of the US population. Although Vizient CDB/RM database reflects the majority of AMCs and many other health care systems nationally, it is inevitable that some cases were not captured in this dataset, particularly if these were managed at or community-based or rural centers that are less likely to report to Vizient. Furthermore, only medical centers reporting for every quarter of the examined time frame (October 2009 to September 2018 for lateral sCSF leak analysis, October 2009 to September 2015 for all sCSF leak and epidural hematoma analysis) were included in this study. Thus, sCSF leak repair rates in the United States are likely higher than our data suggests. Furthermore, as this study is not representative of the entire United States or individual regions, we cannot determine true sCSF leak or repair rates nationally.

Additionally, our study was limited to the time frame of October 2009 to September 2018 due to the reporting mechanisms of Vizient. Our analysis was further complicated by the national transition to *ICD-10* in October 2015. It is known that the approximately 4000 *ICD-9* procedure codes to more than 72,000 *ICD-10* procedure codes and only 5% of the *ICD-9* codes can be linked 1-to-1 with *ICD-10* codes (37). Inconsistencies in identifying *ICD-10* equivalent to CSF rhinorrhea in this coding cohort required exclusion of CSF rhinorrhea *ICD-9* code (349.81) for consistent analysis. This accounts the discrepancy between prior reported sCSF leak repair rates in 2010–2012 and those reported herein (7). More recent data after 2020 is complicated by limited sample size and the COVID-19 epidemic severely reducing patient and surgical encounters. Furthermore, as previously discussed, epidural hematoma data was limited to *ICD-9* diagnostic codes for the purpose of consistency. Increased access to care with more technology—including higher resolution CT imaging—and increased recognition of sCSF leaks may also have contributed to increasing rates of disease identification and repair, however increased awareness alone is unlikely to explain a phenomenon that is now documented to have persisted for more than a decade. Multiple cohort studies have shown increasing incidence of sCSF leaks, supporting a true rise in the incidence in sCSF leak repairs (7,9).

In prior studies, obesity prevalence among sCSF leak repair patients has been as high as 70-80% (5,6). The obesity prevalence of the sCSF leak repair cohort in this national database reviewed was only 32.0%. It is important to note that this is likely an underestimation due to the nature of the data and under-coding.

## CONCLUSIONS

The national rate of for lateral sCSF leaks has increased 110% while no significant increase has been observed in epidural hematoma rates. The rate of all sCSF leak repairs increased by 53% from 2009-2015 and has likely continued to increase. Black patients are at higher-than-expected risk for sCSF leaks, likely due to higher rates of comorbid diseases. The increase in sCSF leaks is observed across the United States in all regions and its rate of increase may surpass the continually increasing rate of obesity. Providers should have a high index of suspicion for sCSF leak, particularly in overweight or obese individuals with multiple related comorbidities.

## FUNDING SOURCES

None declared.

## CONFLICT OF INTEREST STATEMENT

None declared.

## DATA AVAILABILITY STATEMENT

The datasets generated during and/or analyzed during the current study are not publicly available. The datasets generated and analyzed during this study were derived from data in the Vizient Clinical Data Base and Resource Manager, to which most academic institutions report. As such, all queries for study data would need to be approved by Vizient.
